# High-dose sitagliptin for systemic inhibition of dipeptidylpeptidase-4 to enhance engraftment of single cord umbilical cord blood transplantation

**DOI:** 10.18632/oncotarget.22739

**Published:** 2017-11-27

**Authors:** Sherif S. Farag, Robert Nelson, Mitchell S. Cairo, Heather A. O’Leary, Shuhong Zhang, Carol Huntley, David Delgado, Jennifer Schwartz, Mohammad Abu Zaid, Rafat Abonour, Michael Robertson, Hal Broxmeyer

**Affiliations:** ^1^ Division of Hematology and Oncology, Department of Medicine, Indianapolis, Indiana, USA; ^2^ Department of Microbiology and Immunology, Indianapolis, Indiana, USA; ^3^ Department of Pediatrics, Indiana University School of Medicine, Indianapolis, Indiana, USA; ^4^ Indiana University Simon Cancer Center, Indianapolis, Indiana, USA; ^5^ Children and Adolescent Cancer and Blood Diseases Center and Department of Pediatrics, New York Medical College, Valhalla, New York, USA

**Keywords:** cord blood, DPP-4, CD26, engraftment, leukemia

## Abstract

Delayed engraftment remains a limitation of umbilical cord blood (UCB) transplantation. We previously showed that inhibition of dipeptidylpeptidase (DPP)-4 using sitagliptin 600 mg daily was safe with encouraging results on engraftment, but inhibition was not sustained. We evaluated the efficacy and feasibility of higher doses of sitagliptin to enhance engraftment of UCB in patients with hematological cancers. Fifteen patients, median age 41 (range, 18-59) years, received single UCB grafts matched at 4 (n=11) or 5 (n=4) of 6 HLA loci with median nucleated cell dose of 3.5 (range, 2.57-4.57) x10^7^/kg. Sitagliptin 600 mg every 12 hours was administered days -1 to +2. All patients engrafted by day 30, with 12 (80%) engrafting by day 21. The median time to neutrophil engraftment was 19 (range, 12-30) days. Plasma DPP-4 activity was better inhibited with a mean residual trough DPP-4 activity of 70%±19%. Compared to patients previously treated with 600 mg/day, sitagliptin 600 mg every 12 hours appeared to improve engraftment, supporting the hypothesis that more sustained DPP-4 inhibition is required. *In-vivo* inhibition of DPP-4 using high-dose sitagliptin compares favorably with other approaches to enhance UCB engraftment with greater simplicity, and may show synergy in combination with other strategies.

## INTRODUCTION

Umbilical cord blood (UCB) is a source of hematopoietic stem and progenitor cells (HSPC) for transplantation of patients who do not have a human leukocyte antigen (HLA)-matched sibling or adult unrelated donor. However, delayed neutrophil engraftment remains one significant disadvantage of UCB transplantation [1, 2]. Several approaches are being considered to enhance engraftment of UCB, including intraosseous infusion, *ex-vivo* expansion of hematopoietic progenitors, co-transplantation with accessory cells, and enhancing homing of HSPC to the bone marrow [1, 3].

The stromal derived factor (SDF)-1α:CXCR4 axis plays an important role in homing of stem cells [4], and is modulated by the enzyme dipeptidylpeptidase (DPP)-4/CD26 [5]. DPP-4 cleaves the N-terminus dipeptide of SDF-1α, resulting in a truncated-form of SDF-1 that is unable to activate CXCR4; inhibition of DPP-4 enhances migration of CD34+ cells [5]. In pre-clinical models, DPP-4/CD26 inhibition by *ex-vivo* pretreatment of donor HSPC or systemic inhibition using specific inhibitors enhanced engraftment of UCB and murine bone marrow cells [6–8]. Recently, we reported the safety of systemic DPP-4 inhibition using the clinical inhibitor sitagliptin, approved for treatment of type 2 diabetes mellitus, to enhance engraftment of single-unit UCB transplantation [9]. Although times to neutrophil engraftment were encouraging, pharmacodynamic studies suggested the schedule of sitagliptin (600 mg daily) was suboptimal in producing sustained inhibition of plasma DPP-4, which correlated with engraftment [9, 10]. Herein, we present results of a prospective trial of 12-hourly dosing of sitagliptin to enhance neutrophil engraftment of single-unit UCB transplants in patients with high-risk hematological malignancies.

## RESULTS

### Patients and graft characteristics

Fifteen patients were enrolled at Indiana University (n=14) and the New York Medical College (n=1) between January 2013 and July 2016. One patient subsequently found not to have met eligibility because she commenced treatment one day earlier than the prescribed 35-day interval from previous therapy is included in the analysis. The trial was stopped early because of poor accrual. Patients and graft characteristics are summarized in Table 1. All patients received red blood cell-depleted single unit UCB grafts. Notably, 11 of the 15 patients received 4/6 HLA matched UCB units. The median nucleated cell (NC) dose pre-freezing was 3.50 (range, 2.57-4.57) x10^7^/kg, and the median viable NC dose infused was 2.58 (1.28-3.69) x10^7^/kg. Patient characteristics and cell doses infused were similar to those included in our pilot study [9].

**Table 1:** Patient and graft characteristics

	All Patients (N=15)
Age, median (range) years	41 (18-59)
Weight, median (range) kg	82.1 (51.4-133.5)
M/F, n	6/9
Diagnosis, n	
AML	
CR1	7
CR3	1
Primary refractory	2
ALL	
CR1	2
Primary refractory	1
MDS, therapy-related	1
DLBCL, Relapsed and refractory	1
Number of prior chemotherapy cycles, median (range)	3 (1-10)
Days from diagnosis to transplant, median (range)	156 (87-1234)
HLA-match, n	
5 of 6	4
4 of 6	11
UCB graft characteristics:	
Pre-freeze NC, median (range) (x10^7^/kg)	3.50 (2.57-4.57)
Post-thaw viable NC, median (range) (x10^7^/kg)	2.58 (1.28-3.69)
Post-thaw viable cell recovery, median (range) (%)	72 (47-80)
Post-thaw CD34+ cells, median (range) (x10^5^/kg)	2.5 (1.0-7.0)
CFU, median (range) (x10^4^/kg)	2.3 (1.2-7.0)

Abbreviations: AML, acute myeloid leukemia; ALL, acute lymphoblastic leukemia; DLBCL, diffuse large B-cell lymphoma; MDS, myelodysplastic syndrome; CR, complete remission; NC, nucleated cell count; CFU, colony forming units.

### Engraftment outcome

**Figure 1:**
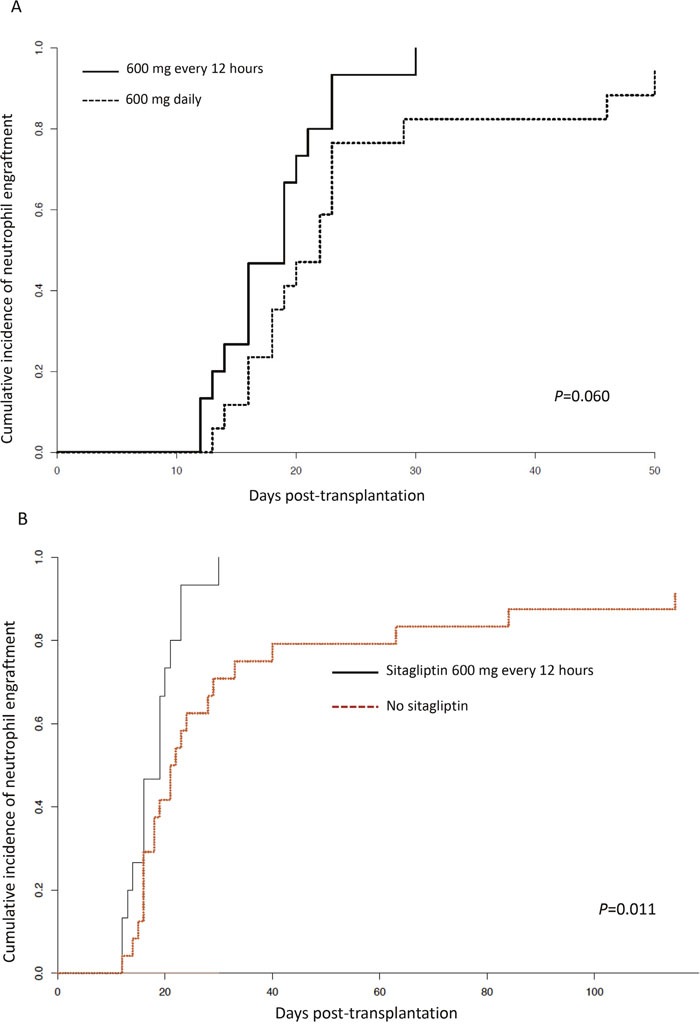
Neutrophil engraftment **(A)** Cumulative incidence of neutrophil engraftment comparing patients receiving sitagliptin 600 mg every 12 hours (n=15) in the current trial with also those receiving 600 mg sitagliptin once daily and red blood cell-depleted UCB units, previously reported (n=17).^9^ **(B)** Cumulative incidence of neutrophil engraftment comparing patients receiving sitagliptin 600 mg every 12 hours (n=15) in the current trial with an institutional control of 24 patients receiving UCB transplants without sitagliptin during a similar time interval (see text for detail).

All 15 patients engrafted by day +30 days following transplantation, with 12 patients engrafting by day +21. The median time to neutrophil engraftment was 19 (range, 12-30) days, with a median 100% (range, 95% - 100%) donor chimerism at engraftment. Figure 1A shows times to neutrophil engraftment of patients treated with sitagliptin 600 mg every 12 hours on the current study compared with the 17 patients also receiving red blood cell-depleted cord blood units but treated with 600 mg daily on our previous trial where the median time to engraftment was 21 (range, 13-50) days (*P*=0.060) [9]. The cumulative incidence of platelet recovery to >20×10^9^/l was 46.7% (95% confidence interval [CI], 19.3% - 74.0%) by day +100, with non-relapse mortality and death from disease relapse as competing risks (40.0% [95% CI, 13.3% - 66.7%]). All engraftment was durable in all patients with no secondary graft failures. As an additional comparison, we compared the engraftment of patients treated with sitagliptin 600 mg every 12 hours on the current study with our institutional cohort of 24 consecutive patients who received UCB transplants for hematological malignancies and a variety of non-malignant disorders (other than hemoglobinopathies) without sitagliptin between January 2011 and December 2016 (Figure 1B). The median age of the latter control patients was 4 (range, 0.4-21) years with 13 males and 11 females, who received UCB grafts that were 4/6 (n=10), 5/6 (n=8), and 6/8 (n=6) HLA-matched and containing a median viable NC dose of 7.2 (3.0-43.9) x10^7^/kg. As shown, despite more than a two and half fold higher median NC dose, engraftment was significantly slower (*P*=0.011) with a median time to engraftment of 21.5 (range, days and a cumulative incidence of engraftment of 87% (95% CI, 72% - 100%) by 100 days post-transplantation.

### Toxicity

Grades 3-4 toxicities were expected and similar to those previously observed [9]. These included mucositis (n=5), self-limiting sinusoidal obstruction syndrome (n=1), thrombotic microangiopathy (n=1), and multiorgan failure due to sepsis (n=2). Blood glucose levels remained essentially unchanged during the sitagliptin dosing period and no episodes of hypoglycemia were observed (data not shown). Indeed, there were no toxicities that could be specifically related to sitagliptin, confirming the safety of the drug even at much higher doses than those used for diabetes mellitus.

The cumulative incidence of non-relapse mortality at 6 months was 46.7% (95% CI 19.9%-73.5%), with death due to relapse/progression of disease a competing risk. Infections, including concurrent adenovirus hepatitis and human herpes virus-6 (n=1), *Escherichia coli* meningitis (n=1), vancomycin-resistant enterococcus sepsis (n=2), hospital-acquired pneumonia (n=1), and aspergillus infection (n=1) were causes of death in 7 patients within the first 180 days, with an additional patient dying on day +217 from *Stenotrophomonas maltophila* sepsis. Non-fatal cytomegalovirus viremia and herpes simplex stomatitis occurred in 4 and 2 patients, respectively. Only one patient developed acute GvHD of the skin, clinical grade 1, during tapering of immunosuppression drugs, which promptly resolved with topical corticosteroid treatment.

### Plasma DDP-4 activity

**Figure 2:**
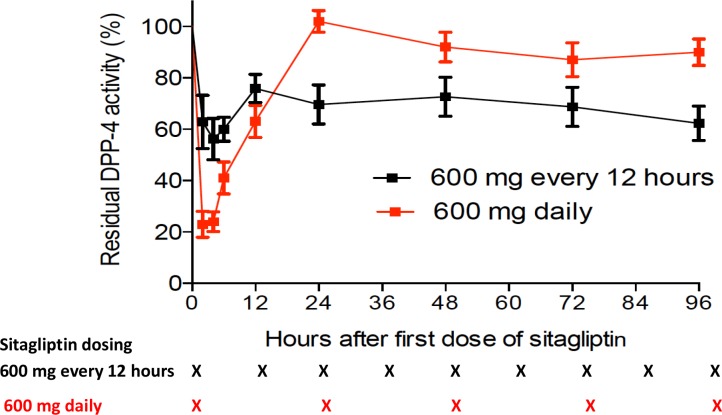
Plasma DPP-4 activity DPP-4 activity following the first following dose of sitagliptin (started on day -1) shown as percentages of baseline, comparing the residual activity in patients treated with sitagliptin 600 mg every 12 hours on the current study with those receiving sitagliptin 600 mg once daily (black curve) and red blood cell depleted UCB transplants, previously reported (red curve).^9^ Times of sitagliptin administration are indicated by “X” at the bottom of the figure. ^*^Indicate trough levels.

The plasma DPP-4 activity was measured at baseline and after dosing, and levels were expressed as residual activity as a percentage of baseline (Figure 2). Maximal inhibition of plasma DPP-4 activity occurred at 2-4 hours after dosing. The mean (± standard error) plasma DPP-4 activity at 4 hours after dosing was 56%±16% of baseline. Inhibition of plasma DPP-4 was better sustained through the dosing interval, with mean residual trough DPP-4 activity of 70%±19%. The median area under the total percent residual DPP-4 activity versus time curve (AUC_A_) for the dosing period was 3,130 (range, 2,004-4,636) h^*^activity, which was significantly lower than that seen in our previous trial using 600 mg sitagliptin daily (median 5,169 [range, 2,932-7,530] h^*^activity; *P*=0.000013) [9]. There was no correlation between maximal inhibition of plasma DPP-4, mean trough levels of DPP-4 activity, or AUC_A_ and engraftment in the current trial (result not shown).

## DISCUSSION

Cell dose is an important determinant of engraftment and long-term outcomes following UCB transplantation [11, 12]. This has led to use of double-unit UCB grafts such that double-unit UCB transplants have exceeded those using single UCB units in recent years [13], without definitive proof of benefit. A recent randomized trial comparing single- versus double-unit UCB transplants did not shown enhanced engraftment or improvement in treatment-related mortality, relapse, or overall survival following double-unit UCB transplants [14]; the authors concluding that single-unit UCB transplants remain a standard of care. We focused on enhancing engraftment of single-unit UCB transplants, as we felt this was the best way to definitively investigate the potential efficacy of DPP-4 inhibition.

The observed kinetics of engraftment in this trial compare quite favorably with other reports where comparable doses and degree of human leukocyte antigen (HLA)-mismatching between UCB unit and recipient were used, as well with other approaches tested to enhance engraftment [15–19], where delays in engraftment still occurs in a proportion of patients and primary graft failure remains problematic. Furthermore, many of the reported approaches to enhance engraftment remain complex, require *ex-vivo* manipulation, and are costly, presenting economic concerns [20], and may be prohibitive for some centers. In our current trial, all patients engrafted neutrophils within 30 days, and 12 of the 15 (80%) patients engrafted before day 21. No graft failures were observed. The higher dose (600 mg twice daily) of sitagliptin used in this trial produced more sustained inhibition of plasma DPP-4, with a median AUC_A_ of 3,130 (range, 2,004-4,636) h^*^activity, significantly lower than that seen in our previous trial using 600 mg sitagliptin daily (median 5,169 [range, 2,932-7,530] h^*^activity; *P*=0.000013) [9]. This may have accounted for the faster engraftment kinetics observed compared to our previously reported pilot trial where plasma DPP-4 inhibition was not sustained with only 600 mg daily of sitagliptin despite similar TNC (median 3.5×10^7^/kg vs 3.61×10^7^/kg, *P*=0.28) and similar CD34 (median 2.6×10^4^/kg vs 1.0×10^4^/kg, *P*=0.27) doses used in patients also receiving red blood cell-depleted UCB units [9], supporting the hypothesis that more sustained DPP-4 inhibition may be required for enhanced engraftment. Unlike in our previously reported study [9], residual AUC_A_ did not correlate with engraftment kinetics in the current study. However, we do not believe that this is necessarily contradictory as it is possible that once below a threshold of residual DPP-4 activity is reached by multiple daily dosing of sitagliptin, further improvement in the speed of engraftment may not be observed. Of note, despite more than a two and a half fold lower infused NC dose, the engraftment kinetics of patients treated on the current study were also significantly faster than an institutional cohort of 24 consecutive patients who received UCB transplants without sitagliptin during the same time interval. As DPP-4 also decreases GM-CSF, G-CSF, interleukin-3 and erythropoietin activities, and inhibition/deletion of DPP-4 enhances their activities and hematopoietic recovery *in vivo* after cytotoxic stress in preclinical studies [6], longer administration of sitagliptin may further enhance engraftment.

The higher dose of sitagliptin was well tolerated. Overall, grades 3-4 toxicities were expected and similar to previously observed [9]. Specifically, there were no episodes of hypoglycemia or toxicity that could be specifically related to sitagliptin, supporting the safety of the drug when administered at much higher doses than those used for diabetes mellitus. Despite earlier neutrophil recovery, however, infections remained the commonest cause of death. Because of the small number of patients included, it is not possible to determine if sitagliptin increased the risk of infection. However, preliminary observations from an ongoing phase II trial (NCT02683525) testing sitagliptin for prevention of graft-versus-host disease following peripheral blood stem cell transplantation from matched related and unrelated donors do not suggest that *in-vivo* DPP-4 inhibition increases the risk of infections (unpublished). Further, our use of ATG in the current trial may have also contributed to the high incidence of infections observed. Notwithstanding, infections are a recognized problem early following UCB transplantation due to delayed immune recovery [21], and highlight the need to improve immune function beyond neutrophil engraftment.

A limitation of our current study is the small number of patients treated. Indeed, the trial failed to meet its accrual goal, in part reflecting the reluctance of clinicians to use single UCB units in adults despite the lack of proven benefit of double UCB units [14]. Importantly, the primary endpoint (engraftment by day +30) satisfied success criteria of the first stage of the trial, and the conditional probability that the null hypothesis would be rejected if the study was continued to full planned accrual is greater than 99%.

Overall, our results indicate that systemic DPP-4 inhibition using high-dose sitagliptin given every 12-hourly enhances engraftment of single-unit UCB transplants, and may offer additional benefits over other strategies in terms of simplicity and lower cost. It is also possible that other strategies may be potentially synergistic with *in vivo* DPP-4 inhibition. In particular, PGE_2_ treatment of donor cells combined with systemic DPP-4 inhibition may be synergistic [22], and also PGE_2_ induces and maintains naïve and memory CD8+ T cells [23], which could improve immune dysfunction following UCB transplantation.

## MATERIALS AND METHODS

### Study design

Eligible patients were aged 18-59 years, had acute myeloid (AML) or lymphoblastic leukemia (ALL), high-risk myelodysplasia, or relapsed chemotherapy-refractory aggressive non-Hodgkin's lymphoma, Karnofsky performance status ≥70%, adequate organ function, and did not have a readily available HLA-matched sibling or volunteer unrelated donor. Patients with diabetes mellitus requiring insulin or oral hypoglycemic agents, and those with a history of pancreatitis, symptomatic cholelithiasis, or a history hypersensitivity to sitagliptin were excluded. Other eligibility criteria were as previously described [9]. The trial was approved by the institutional review boards of Indiana University and New York Medical College.

### Study treatment and selection of UCB graft

The preparative regimen consisted of high-dose melphalan (140 mg/m*^2^* on day -8), thiotepa (10 mg/kg actual body weight on day -7), fludarabine (40 mgm^2^ on days -6 to -3), and rabbit anti-thymocyte globulin (1.5 mg/kg on day -3, and 1.25 mg/kg on days -2 and -1; total dose 4 mg/kg) as previously described [24]. Sitagliptin (Januvia®; Merck & Co., Inc., Whitehouse Station, NJ) 600 mg every 12 hours orally was administered on days -1 through +3; we previously determined in a dose-escalation study that this dose and schedule produced more sustained plasma DPP-4 inhibition without significant toxicity [25]. Graft-versus-host disease (GvHD) prophylaxis used sirolimus and tacrolimus as previously described [9]. Filgrastim (5 μg/kg/day subcutaneously) was started on day +5 and continued until neutrophil recovery. Patients received standard antibiotic prophylaxis using ciprofloxacin, posaconazole and acyclovir against bacterial, mold and viral infections, respectively.

Patients received only single, red blood cell-depleted UCB units that were matched at 4 or more of 6 HLA loci, and contained >2.5 x10^7^/kg NC before freezing [9]. UCB units with the best HLA-match were preferred, and within an HLA match level units with greatest NC numbers were selected.

### Endpoints, sample size and statistical analysis

The primary endpoint of the trial was neutrophil engraftment. The trial was designed to demonstrate an increase in the proportion of patients engrafting by day +30 to ≥70% from an expected 50%, based on results in patients transplanted with single unit UCB units of similar cell doses and degree of HLA-mismatch [26]. Standard definitions of times to neutrophil engraftment and platelet recovery were used [9]. Toxicity was graded using the NCI Common Terminology Criteria for Adverse Events v3.0. Cumulative incidences of neutrophil and platelet engraftment were calculated from day 0 until neutrophil engraftment and platelet recovery, respectively, with death prior to recovery a competing risk. Cumulative incidence of non-relapse mortality (NRM) was calculated from day 0 to death from any cause other than relapse, with death due to relapse a competing risk. Pharmacodynamic studies of DPP-4 activity and calculation of the area under the residual DPP-4 activity versus time curve (AUC_A_) was performed as previously reported [9]. Analyses were performed using SPSS version 22 (IBM Corporation, Armonk, NY), and R version 3.3.0 statistical programs.

## Abbreviations

ALL: acute lymphoblastic leukemia
AML: acute myeloid leukemia
AUC: area under the curve
CFU: colony-forming unit
CR: complete remission
DLBCL: diffuse large B cell lymphoma
DPP-4: dipeptidylpeptidase-4
GvHD: graft-versus-host disease
HLA: human leukocyte antigen
HSPC: hematopoietic stem and progenitor cell
MDS: myelodysplastic syndrome
NC: nucleated cell
SDF-1α: stromal cell derived factor-1α
UCB: umbilical cord blood
